# The genome sequence of the glistening inkcap, *Coprinellus micaceus Coprinellus*; *Coprinellus micaceus* ((Bull.) Vilgalys, Hopple & Jacq. Johnson, 2001)

**DOI:** 10.12688/wellcomeopenres.23349.1

**Published:** 2024-11-12

**Authors:** Richard Wright, Kieran Woof, Royal Botanic Gardens Kew Genome Acquisition Lab, Darwin Tree of Life Barcoding collective, Wellcome Sanger Institute Tree of Life Management, Samples and Laboratory team, Wellcome Sanger Institute Scientific Operations: Sequencing Operations, Wellcome Sanger Institute Tree of Life Core Informatics team, Tree of Life Core Informatics collective, Darwin Tree of Life Consortium

**Affiliations:** 1 Cardiff University, Cardiff, Wales, UK; 2 Royal Botanic Gardens Kew, Richmond, England, UK

**Keywords:** Coprinellus micaceus, glistening inkcap, genome sequence, chromosomal, Agaricales

## Abstract

We present a genome assembly from a specimen of *Coprinellus micaceus* (the glistening inkcap; Basidiomycota; Agaricomycetes; Agaricales; Psathyrellaceae). The genome sequence is 52.0 megabases in span. Most of the assembly is scaffolded into 13 chromosomal pseudomolecules. The mitochondrial genome has also been assembled and is 54.99 kilobases in length.

## Species taxonomy

Eukaryota; Opisthokonta; Fungi; Dikarya; Basidiomycota; Agaricomycotina; Agaricomycetes; Agaricomycetidae; Agaricales; Psathyrellaceae; *Coprinellus*; *Coprinellus micaceus* ((Bull.) Vilgalys, Hopple & Jacq. Johnson, 2001) (NCBI:txid71717)

## Background

*Coprinellus micaceus* (glistening inkcap) is a lamellate agaric that can occasionally grow singly, but more commonly forms dense clusters of sporocarps from deciduous stumps and buried dead wood throughout the year. When young, it has an ochre-brown to yellow-brown, ovoid cap which has a darker shallow umbo at the apex, all of which fades to greyer tones and becomes campanulate to flat in shape, and distinctly radially striate with age, reflecting the gill pattern through the thin pileus flesh ( Levine, 1914). The pileus is enclosed in a white, glistening, granular veil during primordia development (which gives this fungus its specific and common names), which breaks apart into a fine dusting as the pileus expands or rain washes it off. Each cap measures 7–60(–90) mm across. Lamellae are very fine, crowded, pale grey to black with age, deliquescent, and free from attachment to the stipe. The stipe is cylindrical, sometimes widening towards the base, fragile, pruinose, white and slightly browning towards the base, measuring 40–100 × 2–6 mm ( Kibby, 2021; Knudsen & Vesterholt, 2012).

*Coprinellus micaceus* spores are very dark brown, or dark purple to black, smooth, ovoid to mitriform in a plan view, ellipsoid from the side, with a central germ pore smooth, 7.5–10 × 4.5–6 × 4–5.5 μm, producing a very dark spore deposit. Pleurocystidia are few to absent ( Knudsen & Vesterholt, 2012).

In culture, the mycelium is slow growing, cottony to woolly, white, becoming yellow buff in central areas with age. Septa are simple, clamp connections are scarce and obscured by lateral branch origins ( Brodie *et al.*, 1967). This species grows well in culture and can produce sporocarps directly from a range of simple substrates and media ( Humphrey, 1937; Levine, 1914).

*C. micaceus* is known from every continent including Antarctica ( GBIF, 2023). In the UK, it is a very common species wherever there is dead broadleaved woody material, including interior spaces.

The glistening inkcap is known to bioaccumulate metals from its environment, especially boron, cobalt, nickel, potassium, and sodium ( Durkan *et al.*, 2011; Dursun *et al.*, 2006; Tyler, 1980), it has also been partly explored for bioactive compounds ( Zahid *et al.*, 2006).

## Genome sequence report

**Figure 1.**
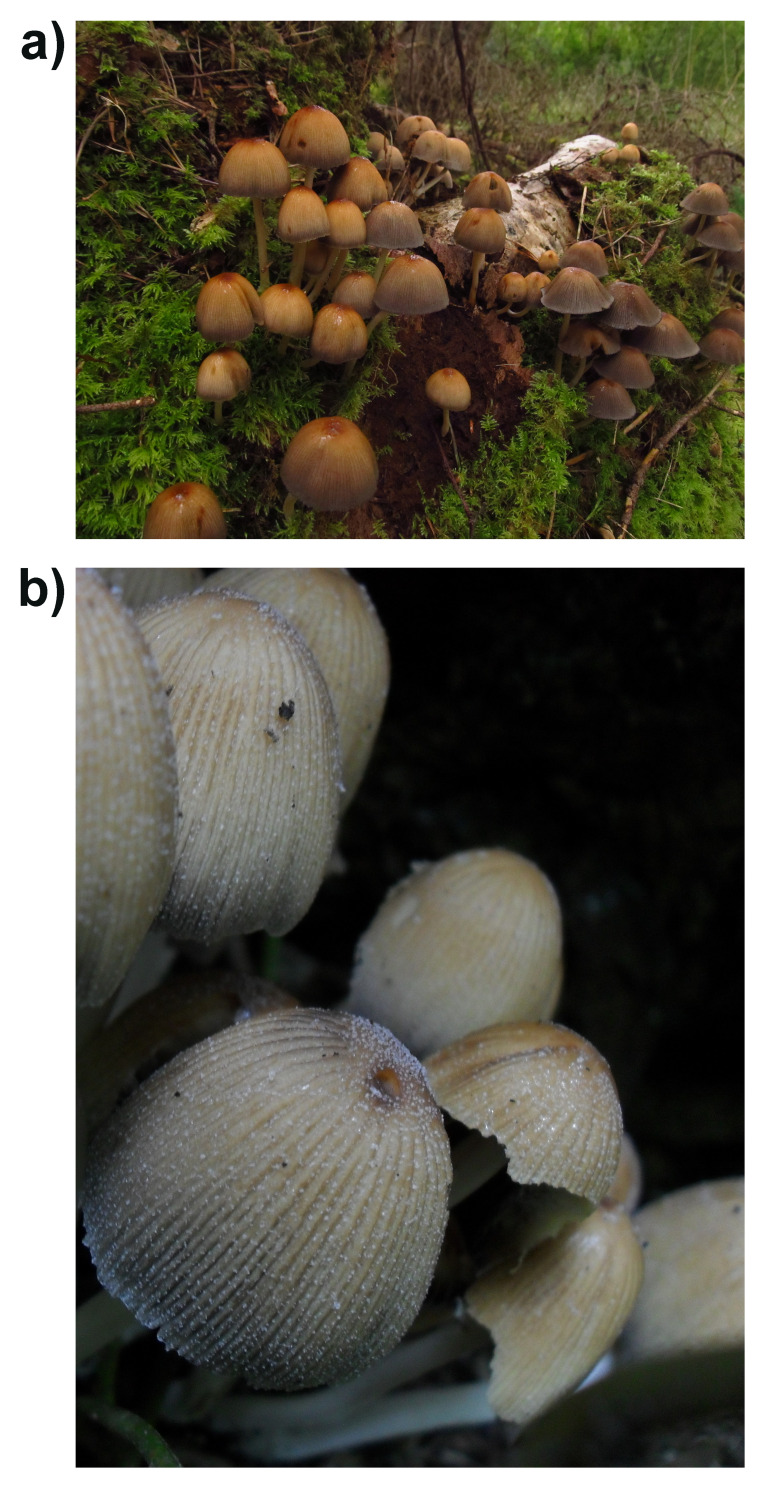
Photographs of the *Coprinellus micaceus* (gfCopMica1) from which samples were taken for sequencing.

The genome was sequenced from a pure culture of *Coprinellus micaceus* ( Figure 1) originally collected from Clyne Woods, Swansea, Wales (51.61, –4.01). A total of 428-fold coverage in Pacific Biosciences single-molecule HiFi long reads was generated. Primary assembly contigs were scaffolded with chromosome conformation Hi-C data. Manual assembly curation corrected 34 missing joins or mis-joins and removed one haplotypic duplication, reducing the scaffold number by 9.17%, and decreasing the scaffold N50 by 4.22%.

**Figure 2.**
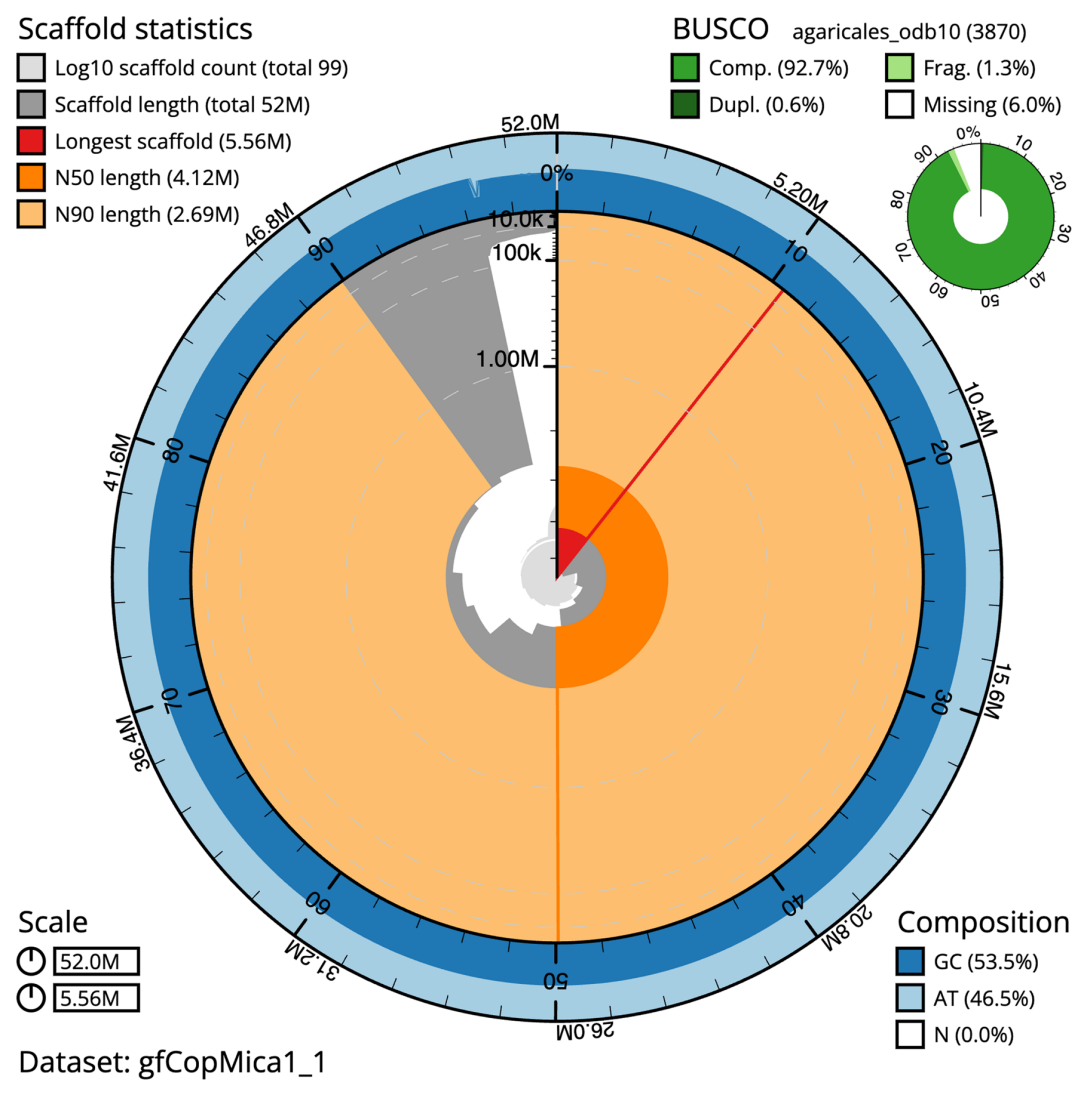
Genome assembly of *Coprinellus micaceus*, gfCopMica1.1: metrics. The BlobToolKit snail plot shows N50 metrics and BUSCO gene completeness. The main plot is divided into 1,000 bins around the circumference with each bin representing 0.1% of the 52,018,476 bp assembly. The distribution of scaffold lengths is shown in dark grey with the plot radius scaled to the longest scaffold present in the assembly (5,557,000 bp, shown in red). Orange and pale-orange arcs show the N50 and N90 scaffold lengths (4,118,337 and 2,692,299 bp), respectively. The pale grey spiral shows the cumulative scaffold count on a log scale with white scale lines showing successive orders of magnitude. The blue and pale-blue area around the outside of the plot shows the distribution of GC, AT and N percentages in the same bins as the inner plot. A summary of complete, fragmented, duplicated and missing BUSCO genes in the agaricales_odb10 set is shown in the top right. An interactive version of this figure is available at https://blobtoolkit.genomehubs.org/view/gfCopMica1_1/dataset/gfCopMica1_1/snail.

**Figure 3.**
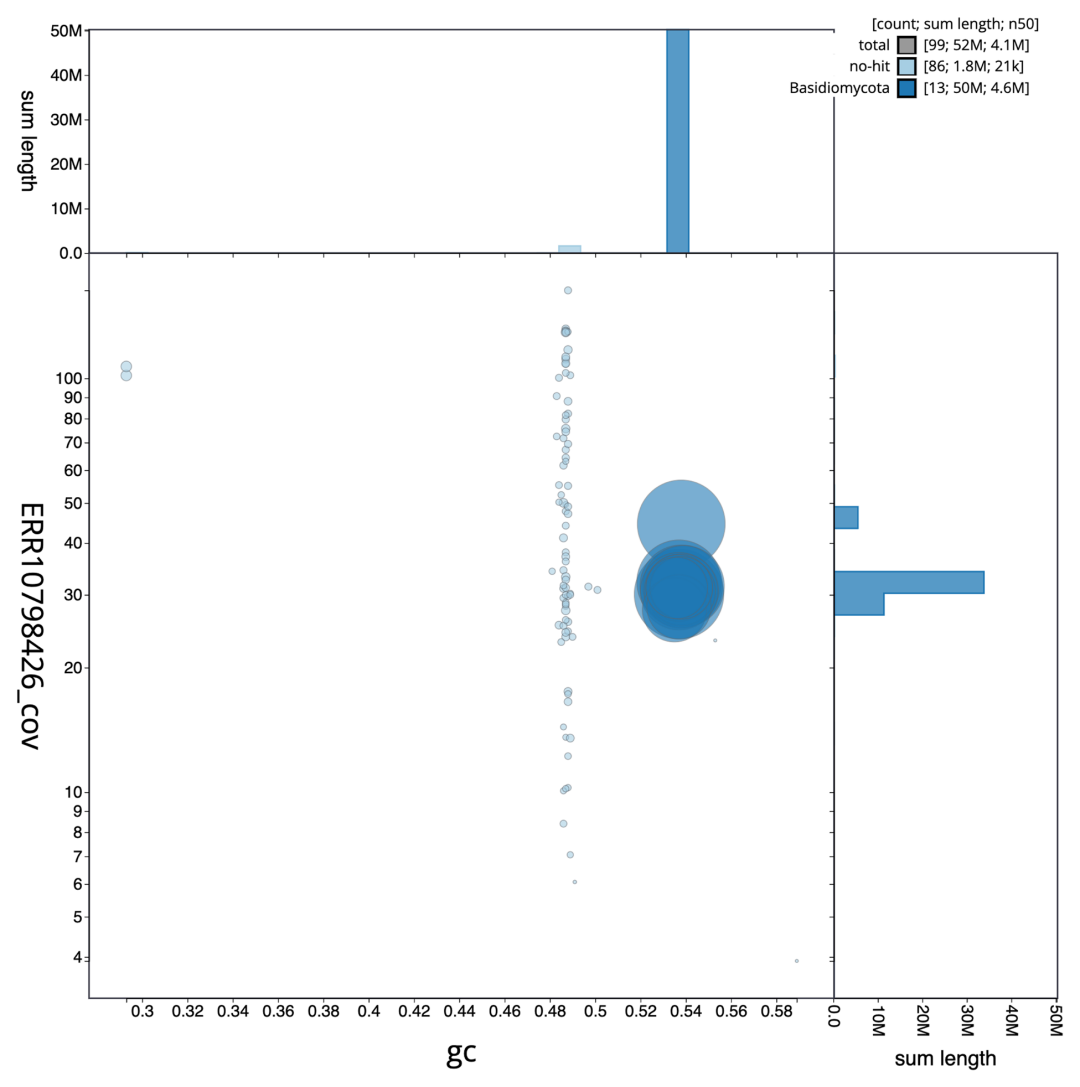
Genome assembly of *Coprinellus micaceus*, gfCopMica1.1: BlobToolKit GC-coverage plot. Sequences are coloured by phylum. Circles are sized in proportion to sequence length. Histograms show the distribution of sequence length sum along each axis. An interactive version of this figure is available at https://blobtoolkit.genomehubs.org/view/gfCopMica1_1/dataset/gfCopMica1_1/blob.

**Figure 4.**
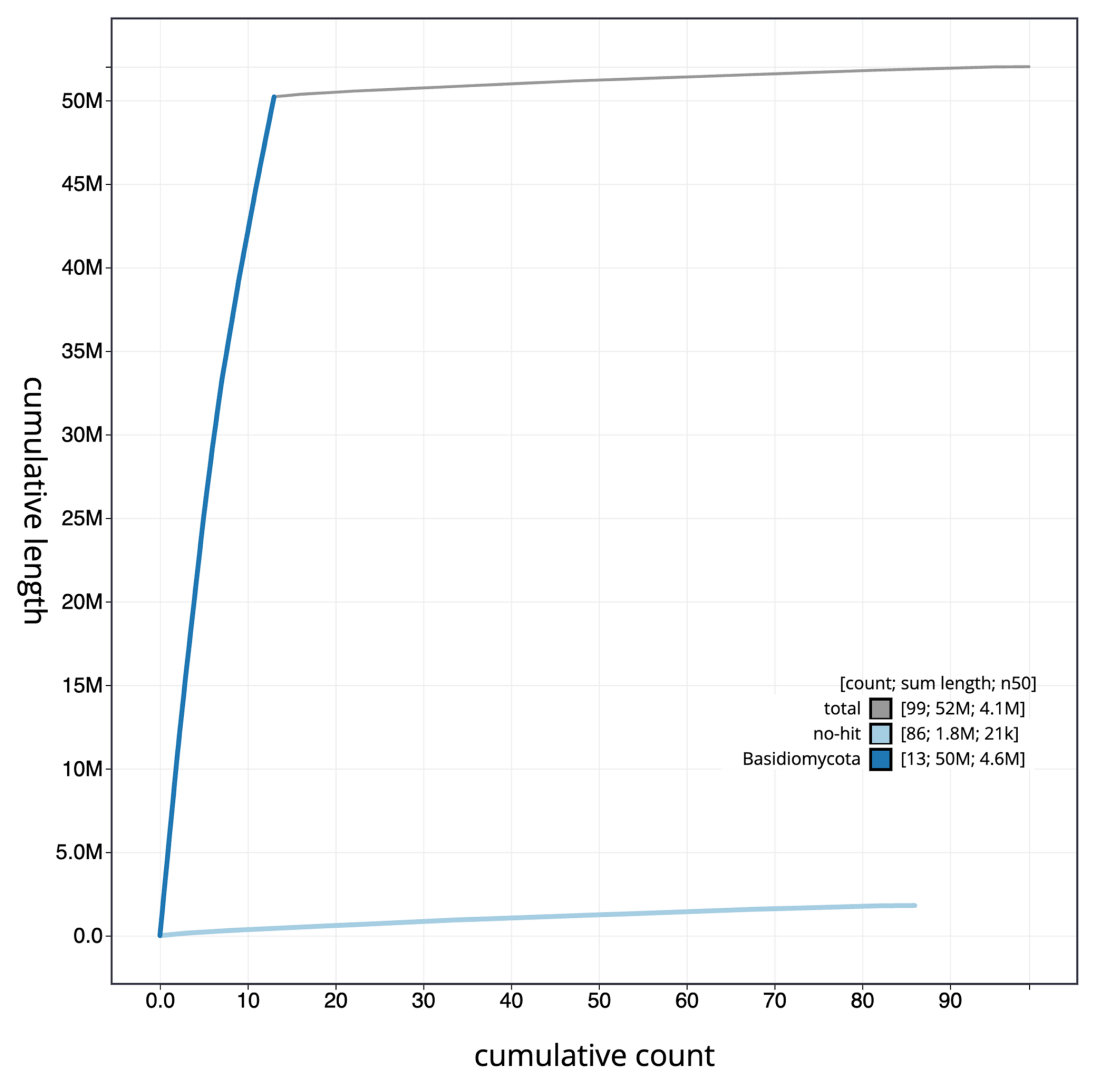
Genome assembly of *Coprinellus micaceus*, gfCopMica1.1: BlobToolKit cumulative sequence plot. The grey line shows cumulative length for all sequences. Coloured lines show cumulative lengths of sequences assigned to each phylum using the buscogenes taxrule. An interactive version of this figure is available at https://blobtoolkit.genomehubs.org/view/gfCopMica1_1/dataset/gfCopMica1_1/cumulative.

**Figure 5.**
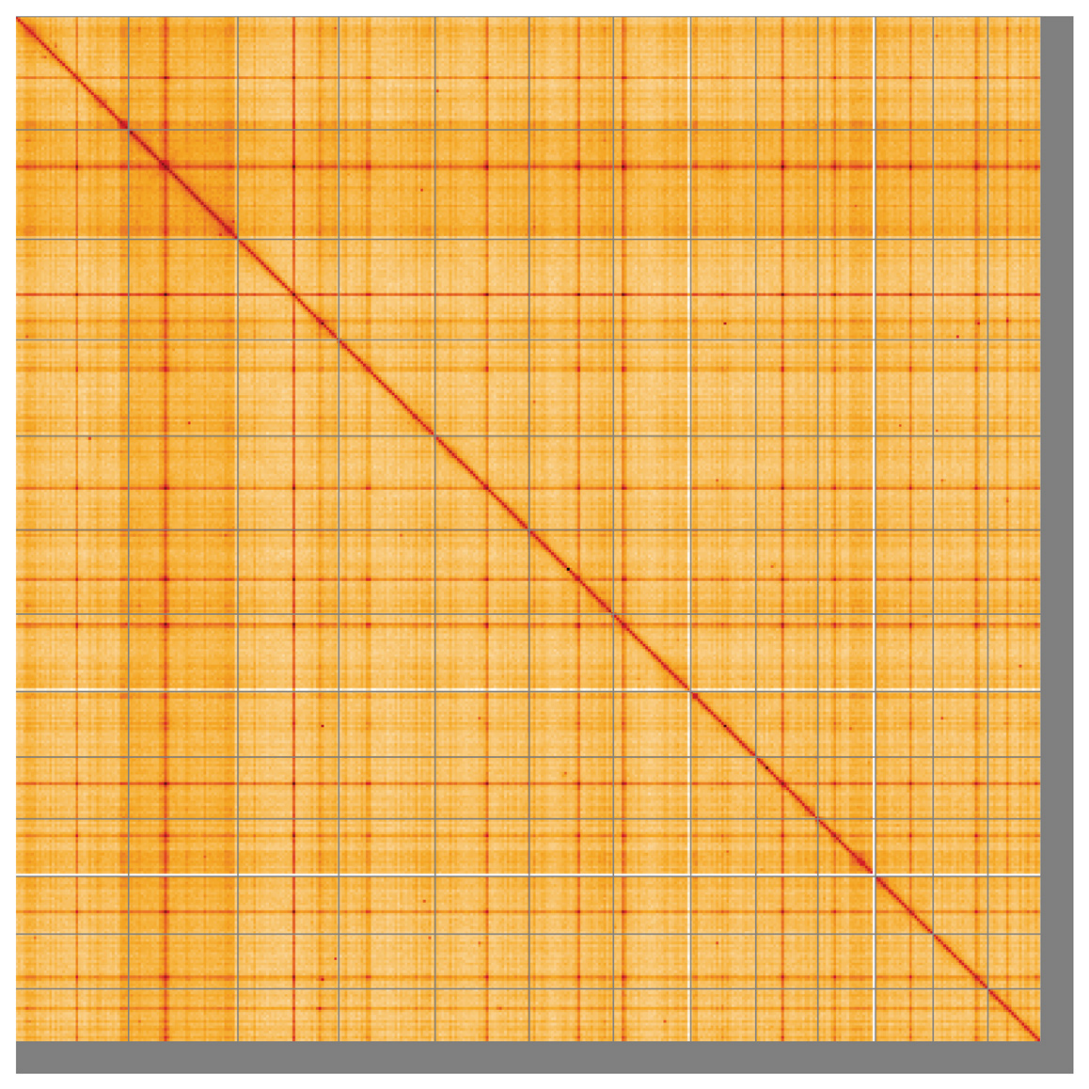
Genome assembly of *Coprinellus micaceus*, gfCopMica1.1: Hi-C contact map of the gfCopMica1.1 assembly, visualised using HiGlass. Chromosomes are shown in order of size from left to right and top to bottom. An interactive version of this figure may be viewed at https://genome-note-higlass.tol.sanger.ac.uk/l/?d=Cwe8vZM3QwSvjttT4xHa3g.

The final assembly has a total length of 52.0 Mb in 98 sequence scaffolds with a scaffold N50 of 4.1 Mb ( Table 1). The snail plot in Figure 2 provides a summary of the assembly statistics, while the distribution of assembly scaffolds on GC proportion and coverage is shown in Figure 3. The cumulative assembly plot in Figure 4 shows curves for subsets of scaffolds assigned to different phyla. Most (96.53%) of the assembly sequence was assigned to 13 chromosomal-level scaffolds. Chromosome-scale scaffolds confirmed by the Hi-C data are named in order of size ( Figure 5; Table 2). While not fully phased, the assembly deposited is of one haplotype. Contigs corresponding to the second haplotype have also been deposited. The mitochondrial genome was also assembled and can be found as a contig within the multifasta file of the genome submission.

**Table 1.** Genome data for *Coprinellus micaceus*, gfCopMica1.1.

Project accession data		
Assembly identifier	gfCopMica1.1	
Species	*Coprinellus micaceus*	
Specimen	gfCopMica1	
NCBI taxonomy ID	71717	
BioProject	PRJEB58952	
BioSample ID	SAMEA9461306	
Isolate information	gfCopMica1	
Assembly metrics *		*Benchmark*
Consensus quality (QV)	62.3	*≥ 40*
*k*-mer completeness	100.0%	*≥ 95%*
BUSCO **	C:92.7%[S:92.1%,D:0.6%], F:1.3%,M:6.0%,n:3,870	*C ≥ 90%*
Percentage of assembly mapped to chromosomes	96.53%	*≥ 90%*
Organelles	Mitochondrial genome: 54.99 kb	*complete single alleles*
Raw data accessions		
PacificBiosciences SEQUEL II	ERR10798426	
Hi-C Illumina	ERR10786027	
Genome assembly		
Assembly accession	GCA_951394405.1	
*Accession of alternate* *haplotype*	GCA_951394415.1	
Span (Mb)	52.0	
Number of contigs	169	
Contig N50 length (Mb)	1.0	
Number of scaffolds	98	
Scaffold N50 length (Mb)	4.1	
Longest scaffold (Mb)	5.56	

* Assembly metric benchmarks are adapted from column VGP-2020 of “Table 1: Proposed standards and metrics for defining genome assembly quality” from Rhie *et al.* (2021).

** BUSCO scores based on the agaricales_odb10 BUSCO set using version 5.3.2. C = complete [S = single copy, D = duplicated], F = fragmented, M = missing, n = number of orthologues in comparison. A full set of BUSCO scores is available at https://blobtoolkit.genomehubs.org/view/gfCopMica1_1/dataset/gfCopMica1_1/busco.

**Table 2.** Chromosomal pseudomolecules in the genome assembly of *Coprinellus micaceus*, gfCopMica1.

INSDC accession	Chromosome	Length (Mb)	GC%
OX596334.1	1	5.56	53.5
OX596335.1	2	5.35	54.0
OX596336.1	3	4.93	53.5
OX596337.1	4	4.72	54.0
OX596338.1	5	4.6	54.0
OX596339.1	6	4.12	53.5
OX596340.1	7	3.79	54.0
OX596341.1	8	3.19	54.0
OX596342.1	9	3.03	53.5
OX596343.1	10	2.82	53.5
OX596344.1	11	2.82	53.5
OX596345.1	12	2.69	54.0
OX596346.1	13	2.59	53.5
OX596347.1	MT	0.05	29.5

The estimated Quality Value (QV) of the final assembly is 62.3 with *k*-mer completeness of 100.0%, and the assembly has a BUSCO v5.3.2 completeness of 92.7% (single = 92.1%, duplicated = 0.6%), using the agaricales_odb10 reference set ( *n* = 3,870).

Metadata for specimens, barcode results, spectra estimates, sequencing runs, contaminants and pre-curation assembly statistics are given at https://links.tol.sanger.ac.uk/species/71717.

## Methods

### Sample acquisition and nucleic acid extraction

The genome was sequenced from a pure culture of *Coprinellus micaceus* (specimen ID KDTOL0000127, ToLID gfCopMica1). The culture was obtained by Richard Wright from spores that had contaminated a sporocarp of *Hydnellum spongiosipes,* collected at Clyne Woods, Swansea, Wales, UK (latitude 51.61, longitude –4.01) by Chris Jones. The sample was snap frozen prior to processing.

The workflow for high molecular weight (HMW) DNA extraction at the Wellcome Sanger Institute (WSI) includes a sequence of core procedures: sample preparation; sample homogenisation, DNA extraction, fragmentation, and clean-up. In sample preparation, the gfCopMica1 sample was weighed and dissected on dry ice ( Jay *et al.*, 2023). For sample homogenisation, mycelium tissue was cryogenically disrupted using the Covaris cryoPREP ^®^ Automated Dry Pulverizer ( Narváez-Gómez *et al.*, 2023). HMW DNA was extracted using the Automated MagAttract v1 protocol ( Sheerin *et al.*, 2023). DNA was sheared into an average fragment size of 12–20 kb in a Megaruptor 3 system with speed setting 30 ( Todorovic *et al.*, 2023). Sheared DNA was purified by solid-phase reversible immobilisation ( Strickland *et al.*, 2023): in brief, the method employs a 1.8X ratio of AMPure PB beads to sample to eliminate shorter fragments and concentrate the DNA. The concentration of the sheared and purified DNA was assessed using a Nanodrop spectrophotometer and Qubit Fluorometer and Qubit dsDNA High Sensitivity Assay kit. Fragment size distribution was evaluated by running the sample on the FemtoPulse system.

Protocols developed by the WSI Tree of Life laboratory are publicly available on protocols.io ( Denton *et al.*, 2023).

### Sequencing

Pacific Biosciences HiFi circular consensus DNA sequencing libraries were constructed according to the manufacturers’ instructions. DNA sequencing was performed by the Scientific Operations core at the WSI on a Pacific Biosciences SEQUEL II instrument. Hi-C data were also generated from mycelium tissue of gfCopMica1 using the Arima2 kit and sequenced on the Illumina NovaSeq 6000 instrument.

### Genome assembly, curation and evaluation

Assembly was carried out with Hifiasm ( Cheng *et al.*, 2021) and haplotypic duplication was identified and removed with purge_dups ( Guan *et al.*, 2020). The assembly was then scaffolded with Hi-C data ( Rao *et al.*, 2014) using YaHS ( Zhou *et al.*, 2023). The assembly was checked for contamination and corrected using the gEVAL system ( Chow *et al.*, 2016) as described previously ( Howe *et al.*, 2021). Manual curation was performed using gEVAL, HiGlass ( Kerpedjiev *et al.*, 2018) and PretextView ( Harry, 2022). The mitochondrial genome was assembled using MitoHiFi ( Uliano-Silva *et al.*, 2023), which runs MitoFinder ( Allio *et al.*, 2020) and uses these annotations to select the final mitochondrial contig and to ensure the general quality of the sequence.

A Hi-C map for the final assembly was produced using bwa-mem2 ( Vasimuddin *et al.*, 2019) in the Cooler file format ( Abdennur & Mirny, 2020). To assess the assembly metrics, the *k*-mer completeness and QV consensus quality values were calculated in Merqury ( Rhie *et al.*, 2020). This work was done using Nextflow ( Di Tommaso *et al.*, 2017) DSL2 pipelines “sanger-tol/readmapping” ( Surana *et al.*, 2023a) and “sanger-tol/genomenote” ( Surana *et al.*, 2023b). The genome was analysed within the BlobToolKit environment ( Challis *et al.*, 2020) and BUSCO scores ( Manni *et al.*, 2021; Simão *et al.*, 2015) were calculated.

Table 3 contains a list of relevant software tool versions and sources.

**Table 3.** Software tools: versions and sources.

Software tool	Version	Source
BlobToolKit	4.1.7	https://github.com/blobtoolkit/blobtoolkit
BUSCO	5.3.2	https://gitlab.com/ezlab/busco
gEVAL	N/A	https://geval.org.uk/
Hifiasm	0.16.1-r375	https://github.com/chhylp123/hifiasm
HiGlass	1.11.6	https://github.com/higlass/higlass
Merqury	MerquryFK	https://github.com/thegenemyers/MERQURY.FK
MitoHiFi	2	https://github.com/marcelauliano/MitoHiFi
PretextView	0.2	https://github.com/wtsi-hpag/PretextView
purge_dups	1.2.3	https://github.com/dfguan/purge_dups
sanger-tol/ genomenote	v1.0	https://github.com/sanger-tol/genomenote
sanger-tol/ readmapping	1.1.0	https://github.com/sanger-tol/readmapping/tree/1.1.0
YaHS	1.2a	https://github.com/c-zhou/yahs

### Wellcome Sanger Institute – Legal and Governance

The materials that have contributed to this genome note have been supplied by a Darwin Tree of Life Partner. The submission of materials by a Darwin Tree of Life Partner is subject to the **‘Darwin Tree of Life Project Sampling Code of Practice’**, which can be found in full on the Darwin Tree of Life website here. By agreeing with and signing up to the Sampling Code of Practice, the Darwin Tree of Life Partner agrees they will meet the legal and ethical requirements and standards set out within this document in respect of all samples acquired for, and supplied to, the Darwin Tree of Life Project.

Further, the Wellcome Sanger Institute employs a process whereby due diligence is carried out proportionate to the nature of the materials themselves, and the circumstances under which they have been/are to be collected and provided for use. The purpose of this is to address and mitigate any potential legal and/or ethical implications of receipt and use of the materials as part of the research project, and to ensure that in doing so we align with best practice wherever possible. The overarching areas of consideration are:

•   Ethical review of provenance and sourcing of the material

•   Legality of collection, transfer and use (national and international)

Each transfer of samples is further undertaken according to a Research Collaboration Agreement or Material Transfer Agreement entered into by the Darwin Tree of Life Partner, Genome Research Limited (operating as the Wellcome Sanger Institute), and in some circumstances other Darwin Tree of Life collaborators.

## Data Availability

European Nucleotide Archive: *Coprinellus micaceus*. Accession number PRJEB58952; https://identifiers.org/ena.embl/PRJEB58952. The genome sequence is released openly for reuse. The *Coprinellus micaceus* genome sequencing initiative is part of the Darwin Tree of Life (DToL) project. All raw sequence data and the assembly have been deposited in INSDC databases. The genome will be annotated using available RNA-Seq data and presented through the Ensembl pipeline at the European Bioinformatics Institute. Raw data and assembly accession identifiers are reported in Table 1.

## Author information

Members of the Royal Botanic Gardens Kew Genome Acquisition Lab are listed here: https://doi.org/10.5281/zenodo.4786680.

Members of the Darwin Tree of Life Barcoding collective are listed here: https://doi.org/10.5281/zenodo.4893703.

Members of the Wellcome Sanger Institute Tree of Life Management, Samples and Laboratory team are listed here: https://doi.org/10.5281/zenodo.10066175.

Members of Wellcome Sanger Institute Scientific Operations: Sequencing Operations are listed here: https://doi.org/10.5281/zenodo.10043364.

Members of the Wellcome Sanger Institute Tree of Life Core Informatics team are listed here: https://doi.org/10.5281/zenodo.10066637.

Members of the Tree of Life Core Informatics collective are listed here: https://doi.org/10.5281/zenodo.5013541.

Members of the Darwin Tree of Life Consortium are listed here: https://doi.org/10.5281/zenodo.4783558.

## References

[ref-1] Abdennur N Mirny LA : Cooler: scalable storage for Hi-C data and other genomically labeled arrays. *Bioinformatics.* 2020;36(1):311–316. 10.1093/bioinformatics/btz540 31290943 PMC8205516

[ref-2] Allio R Schomaker-Bastos A Romiguier J : MitoFinder: efficient automated large-scale extraction of mitogenomic data in target enrichment phylogenomics. *Mol Ecol Resour.* 2020;20(4):892–905. 10.1111/1755-0998.13160 32243090 PMC7497042

[ref-3] Brodie HJ Bandoni RJ Cole KM : Genetic studies in the genus coprinus.1967. 10.14288/1.0105255

[ref-4] Challis R Richards E Rajan J : BlobToolKit – interactive quality assessment of genome assemblies. *G3 (Bethesda).* 2020;10(4):1361–1374. 10.1534/g3.119.400908 32071071 PMC7144090

[ref-5] Cheng H Concepcion GT Feng X : Haplotype-resolved *de novo* assembly using phased assembly graphs with hifiasm. *Nat Methods.* 2021;18(2):170–175. 10.1038/s41592-020-01056-5 33526886 PMC7961889

[ref-6] Chow W Brugger K Caccamo M : gEVAL — a web-based browser for evaluating genome assemblies. *Bioinformatics.* 2016;32(16):2508–2510. 10.1093/bioinformatics/btw159 27153597 PMC4978925

[ref-7] Denton A Yatsenko H Jay J : Sanger Tree of Life wet laboratory protocol collection V.1. *protocols.io.* 2023. 10.17504/protocols.io.8epv5xxy6g1b/v1

[ref-8] Di Tommaso P Chatzou M Floden EW : Nextflow enables reproducible computational workflows. *Nat Biotechnol.* 2017;35(4):316–319. 10.1038/nbt.3820 28398311

[ref-9] Durkan N Dogan Y Unver MC : Concentrations of trace elements aluminum, boron, cobalt and tin in various wild edible mushroom species from Buyuk Menderes River Basin of Turkey by ICP-OES Metal Accumulation in Crops and Potential Health Risk View project. *Trace Elements and Electrolytes.* 2011;28:242–248. 10.5414/TEX01198

[ref-10] Dursun N Özcan MM Kaşik G : Mineral contents of 34 species of edible mushrooms growing wild in Turkey. *J Sci Food Agric.* 2006;86(7):1087–1094. 10.1002/jsfa.2462

[ref-11] GBIF: GBIF Occurrence Download - Coprinellus micaceus. *GBIF Occurrence Download*.2023; [Accessed 28 August 2023]. 10.15468/dl.kvnd5f

[ref-12] Guan D McCarthy SA Wood J : Identifying and removing haplotypic duplication in primary genome assemblies. *Bioinformatics.* 2020;36(9):2896–2898. 10.1093/bioinformatics/btaa025 31971576 PMC7203741

[ref-13] Harry E : PretextView (Paired REad TEXTure Viewer): a desktop application for viewing pretext contact maps.2022. Reference Source

[ref-14] Howe K Chow W Collins J : Significantly improving the quality of genome assemblies through curation. *GigaScience.* 2021;10(1): giaa153. 10.1093/gigascience/giaa153 33420778 PMC7794651

[ref-15] Humphrey SS : Note on the production of fruiting bodies of Coprinus micaceus in culture. *Ohio J Sci.* 1937;37(1):62–64. Reference Source

[ref-16] Jay J Yatsenko H Narváez-Gómez JP : Sanger Tree of Life sample preparation: triage and dissection. *protocols.io.* 2023. 10.17504/protocols.io.x54v9prmqg3e/v1

[ref-17] Kerpedjiev P Abdennur N Lekschas F : HiGlass: web-based visual exploration and analysis of genome interaction maps. *Genome Biol.* 2018;19(1): 125. 10.1186/s13059-018-1486-1 30143029 PMC6109259

[ref-18] Kibby G : Mushrooms and toadstools of Britain & Europe.Agarics. Part 2.2021;3 Reference Source

[ref-19] Knudsen H Vesterholt J : Funga Nordica.2nd ed., Nordsvamp.2012;2 Reference Source

[ref-20] Levine M : The origin and development of the Lamellae in Coprinus micaceus. *Amer J Bot.* 1914;1(7):343–356. 10.2307/2435139

[ref-21] Manni M Berkeley MR Seppey M : BUSCO update: novel and streamlined workflows along with broader and deeper phylogenetic coverage for scoring of eukaryotic, prokaryotic, and viral genomes. *Mol Biol Evol.* 2021;38(10):4647–4654. 10.1093/molbev/msab199 34320186 PMC8476166

[ref-22] Narváez-Gómez JP Mbye H Oatley G : Sanger Tree of Life sample homogenisation: covaris cryoPREP ^®^ automated dry pulverizer V.1. *protocols.io.* 2023. 10.17504/protocols.io.eq2lyjp5qlx9/v1

[ref-23] Rao SSP Huntley MH Durand NC : A 3D map of the human genome at kilobase resolution reveals principles of chromatin looping. *Cell.* 2014;159(7):1665–1680. 10.1016/j.cell.2014.11.021 25497547 PMC5635824

[ref-24] Rhie A McCarthy SA Fedrigo O : Towards complete and error-free genome assemblies of all vertebrate species. *Nature.* 2021;592(7856):737–746. 10.1038/s41586-021-03451-0 33911273 PMC8081667

[ref-25] Rhie A Walenz BP Koren S : Merqury: reference-free quality, completeness, and phasing assessment for genome assemblies. *Genome Biol.* 2020;21(1): 245. 10.1186/s13059-020-02134-9 32928274 PMC7488777

[ref-26] Sheerin E Sampaio F Oatley G : Sanger Tree of Life HMW DNA extraction: automated MagAttract v.1. *protocols.io.* 2023. 10.17504/protocols.io.x54v9p2z1g3e/v1

[ref-27] Simão FA Waterhouse RM Ioannidis P : BUSCO: assessing genome assembly and annotation completeness with single-copy orthologs. *Bioinformatics.* 2015;31(19):3210–3212. 10.1093/bioinformatics/btv351 26059717

[ref-28] Strickland M Cornwell C Howard C : Sanger Tree of Life fragmented DNA clean up: manual SPRI. *protocols.io.* 2023. 10.17504/protocols.io.kxygx3y1dg8j/v1

[ref-29] Surana P Muffato M Qi G : Sanger-tol/readmapping: sanger-tol/readmapping v1.1.0 - Hebridean Black (1.1.0). *Zenodo.* 2023a. 10.5281/zenodo.7755669

[ref-30] Surana P Muffato M Sadasivan Baby C : sanger-tol/genomenote (v1.0.dev). *Zenodo.* 2023b. 10.5281/zenodo.6785935

[ref-31] Todorovic M Sampaio F Howard C : Sanger Tree of Life HMW DNA fragmentation: diagenode Megaruptor ^®^3 for PacBio HiFi. *protocols.io.* 2023. 10.17504/protocols.io.8epv5x2zjg1b/v1

[ref-32] Tyler G : Metals in sporophores of basidiomycetes. *Trans Brit Mycol Soc.* 1980;74(1):41–49. 10.1016/S0007-1536(80)80005-2

[ref-33] Uliano-Silva M Ferreira JGRN Krasheninnikova K : MitoHiFi: a python pipeline for mitochondrial genome assembly from PacBio high fidelity reads. *BMC Bioinformatics.* 2023;24(1): 288. 10.1186/s12859-023-05385-y 37464285 PMC10354987

[ref-34] Vasimuddin M Misra S Li H : Efficient architecture-aware acceleration of BWA-MEM for multicore systems.In: *2019 IEEE International Parallel and Distributed Processing Symposium (IPDPS).*IEEE,2019;314–324. 10.1109/IPDPS.2019.00041

[ref-35] Zahid S Udenigwe CC Ata A : New bioactive natural products from *Coprinus micaceus*. *Nat Prod Res.* 2006;20(14):1283–1289. 10.1080/14786410601101829 17393652

[ref-36] Zhou C McCarthy SA Durbin R : YaHS: yet another Hi-C scaffolding tool. *Bioinformatics.* 2023;39(1): btac808. 10.1093/bioinformatics/btac808 36525368 PMC9848053

