# Supplementary material for: The genome sequence of the glistening inkcap, Coprinellus micaceus Coprinellus; Coprinellus micaceus ((Bull.) Vilgalys, Hopple & Jacq. Johnson, 2001)
Source: Wellcome Open Res. 2024 Nov 12;9:677. [Version 1] doi: 10.12688/wellcomeopenres.23349.1 (PMC11617826; doi:10.12688/wellcomeopenres.23349.1)
Supplement: Peer review history

# Reviewer report: Reviewer response for version 1

**DOI:** 10.21956/wellcomeopenres.25734.r112405

**Published:** 2024-12-04

**Authors:** Erich M. Schwarz

**Affiliations:** 1 Cornell University, Ithaca, New York, USA

This report is well and convincingly written, with a concise but substantial description of the organism's significance and an equally concise but clear description of generation of the genome assembly.  I have only a few comments:

 1. In the Background:

 1a. The authors write "during primordia development"; shouldn't this instead be written "during primordium development"?

 1b. The authors write, "mitriform in a plan view"; what is a 'plan view'?  Could this be reworded to be clearer?

 1c. The authors write, "with a central germ pore smooth"; is this really what they wanted to write?  Or did they mean to write "with a smooth central germ pore"?

 2. In Data Availability, the authors write, "The genome will be annotated using available RNA-Seq data and presented through the Ensembl pipeline at the European Bioinformatics Institute."

 2a. *What* 'available RNA-Seq data'?  Is there already an accession number for these data?  Was it published somewhere?  If the authors themselves generated it, why is it not at least mentioned in this paper except here?

 2b. What is the timetable for this annotation happening, and by whom?  I've seen a number of these claims now in various Tree of Life papers and they leave me wondering when the gene predictions will actually be done.  If the pipeline can be done quickly and automatically, wouldn't it make sense to add the protein-coding gene predictions (and appropriate coauthors) to *this* paper?

Are sufficient details of methods and materials provided to allow replication by others?

Yes

Is the rationale for creating the dataset(s) clearly described?

Yes

Are the datasets clearly presented in a useable and accessible format?

Yes

Are the protocols appropriate and is the work technically sound?

Yes

Reviewer Expertise:

Genome assembly, annotation, and analysis (primarily in nematodes, but with some work in plants and fungi).

I confirm that I have read this submission and believe that I have an appropriate level of expertise to confirm that it is of an acceptable scientific standard.

# Reviewer report: Reviewer response for version 1

**DOI:** 10.21956/wellcomeopenres.25734.r111522

**Published:** 2024-11-29

**Authors:** Bhagya C.Thimmappa

**Affiliations:** 1 ARS, USDA, Chatsworth, NJ, USA

The authors report the nuclear and mitochondrial genome assembly for *Coprinellus micaceus *in this work. The nuclear genome is assembled into 13 chromosomal pseudomolecules with a length of 52.0 megabases.

 The nuclear and mitochondrial genome assemblies reported here are valuable for comparative and evolutionary research on *Coprinellus *and closely related species. The authors provide a clear introduction, and the manuscript reads well with a well-documented method section. Further, one could benefit from nuclear and mitochondrial genome annotation.

 Minor comments:

1. Please explain “lamellate agaric *”* for a broader audience.
2. How do the genomic features of *C. micaceus* vary across different continents?

Are sufficient details of methods and materials provided to allow replication by others?

Yes

Is the rationale for creating the dataset(s) clearly described?

Yes

Are the datasets clearly presented in a useable and accessible format?

Yes

Are the protocols appropriate and is the work technically sound?

Yes

Reviewer Expertise:

Bioinformatics, Genomics, Multi-Omics, Mycology

I confirm that I have read this submission and believe that I have an appropriate level of expertise to confirm that it is of an acceptable scientific standard.
